# Crossover Trial of Pemafibrate and Omega-3-Acid Ethyl Esters for Hypertriglyceridemia in Patients with Cardiovascular Disease

**DOI:** 10.31662/jmaj.2025-0177

**Published:** 2025-12-05

**Authors:** Akira Sezai, Makoto Taoka, Hisakuni Sekino, Masashi Tanaka

**Affiliations:** 1 Department of Cardiovascular Surgery, Nihon University School of Medicine, Tokyo, Japan; 2 Sekino Hospital, Tokyo, Japan

**Keywords:** hypertriglyceridemia, triglyceride, pemafibrate, fibrate, omega-3-acid ethyl esters, EPA, DHA

## Abstract

**Introduction:**

Statins can treat dyslipidemia, but even if low-density lipoprotein decreases to target levels, high triglyceride (TG) levels may represent a residual risk. Therefore, we performed a crossover study comparing pemafibrate and omega-3-acid ethyl esters docosahexaenoic acid (DHA) and eicosapentaenoic acid (EPA) in patients with untreated hypertriglyceridemia.

**Methods:**

Patients were randomized by the envelope method to pemafibrate or DHA+EPA for 6 months and then switched to the other medication for 6 months. The primary endpoint was TG level, and secondary endpoints were lipid markers, fatty acid 4-fractionation, kidney and liver markers, and the Fibrosis-4 index.

**Results:**

In the 36 analyzed patients, pemafibrate showed a significantly greater decrease in TG (p < 0.001) and remnant-like particles cholesterol (p = 0.001) and a significantly greater increase in high-density lipoprotein (p < 0.001), but DHA+EPA showed a significantly greater improvement in fatty acid 4-fractionation (p < 0.001).

**Conclusions:**

When combined with a statin, pemafibrate appears to have a stronger effect in lowering TG and remnant-like particles cholesterol but DHA+EPA appears to be more effective in terms of fatty acids. Pemafibrate may be an effective first choice for hypertriglyceridemia, and add-on DHA+EPA may be beneficial when pemafibrate is not sufficiently effective. Findings need to be confirmed in larger studies.

## Introduction

Statins are used to treat dyslipidemia, but even if low-density lipoprotein (LDL) levels decrease to target values, high triglyceride (TG) levels may represent a residual risk. A large-scale meta-analysis of clinical trials involving 374,358 people taking drugs with TG-lowering effects found that a 1-nmol/L decrease in TG decreased the incidence of major cardiovascular events by 16%, which was approximately equivalent to the 20% reduction of LDL-cholesterol (LDL-C) ^(1)^. A meta-analysis of the incidence of cerebrovascular and cardiovascular events in patients with hypertriglyceridemia or hypertriglyceridemia and low high-density lipoprotein (HDL) levels who received TG-lowering therapy (fibrates, nicotinic acid derivatives, omega-3 fatty acids) was conducted. In this meta-analysis, it was reported that TG-lowering therapy reduced the incidence of cerebrovascular and cardiovascular events by 18% in patients with hypertriglyceridemia and by 29% in patients with hypertriglyceridemia and low HDL levels, regardless of whether or not they were taking statins ^(2)^. High TG levels have been reported to be associated with coronary artery disease risk in Japan, and in a 10-year follow-up study to identify factors associated with coronary artery disease in 6,966 middle-aged Japanese men, TG was identified as an independent risk factor for coronary artery disease ^(3)^. In an 18-year follow-up study of 9,887 subjects, high blood pressure, low HDL cholesterol, and high TG were risk factors for ischemic heart disease and stroke ^(4)^. In an observational study of 1,771 type 2 diabetic patients with no previous cardiovascular disease over 7.9 years, high TG (p < 0.01) was a risk factor for coronary artery disease events and stroke events, along with high LDL (p < 0.01), male gender (p = 0.02), and high hemoglobin A1c (HbA1c) (p = 0.05) ^(5)^, and the importance of TG-lowering therapy is evident in many clinical studies. Drugs that lower TG include fibrates, nicotinic acid derivatives, and omega-3 fatty acids. Recently, pemafibrate, a selective peroxisome proliferator-activated receptor alpha (PPARα) modulator that activates PPARα with higher selectivity than conventional drugs, was reported to have a stronger effect in lowering TG and raising HDL levels than conventional fibrate drugs (PROMINENT study) ^(6)^. Furthermore, conventional fibrate drugs are contraindicated in patients with moderate renal impairment (serum creatinine level ^3^ 2.5 mg/dL) because these drugs are metabolized in the kidneys; however, pemafibrate is not metabolized by the kidneys and therefore can be used also in patients with renal impairment. The authors previously performed a comparative study of the omega-3 fatty acids eicosapentaenoic acid (EPA), and EPA+docosahexaenoic acid (DHA) in patients with dyslipidemia who were taking statins and had TG levels of 150 mg/dL or higher ^(7)^.

Statins can treat dyslipidemia, but even if LDL decreases to target levels, high TG levels may represent a residual risk. Therefore, we performed a crossover study comparing pemafibrate and omega-3-acid ethyl esters (EPA+DHA) in patients with untreated hypertriglyceridemia.

## Materials and Methods

### Study protocol

Participants were outpatients at Sekino Hospital (a logistical support hospital of Nihon University Itabashi Hospital), Tokyo, Japan, who had cardiovascular disease and untreated hypertriglyceridemia (TG level, ^3^ 150 mg/dL, after overnight fasting) and were taking statins. Patients were randomized by the envelope method to receive treatment with either pemafibrate (Kowa Pharmaceutical Company Limited., Tokyo, Japan) or omega-3-acid ethyl esters (DHA+EPA, Takeda Pharmaceutical Company Ltd., Osaka, Japan) for 6 months (first stage). After 6 months of treatment, patients who were assigned to pemafibrate were switched to DHA+EPA, while those who were assigned to DHA+EPA were switched to pemafibrate. The second stage treatment duration lasted for 6 months. For ethical reasons, no washout period was scheduled. The package insert of pemafibrate (Kowa Pharmaceutical Company Ltd.) indicates that the half-life is about 2 hours. In contrast, there is no information about the half-life of DHA+EPA. Therefore, in this study, the efficacy assessment was conducted at the completion of the 6 months of treatment in which there is no influence by prior therapy. Pemafibrate was administered at a dose of 0.1 mg twice daily (after breakfast and dinner), and DHA+EPA was administered at a dose of 2 g once daily (after breakfast). During the study period, the TG level was measured at monthly intervals. If the level was 150 mg/dL or higher, the dose of pemafibrate was increased by 0.2 mg/day or that of EPA+DHA was increased by 2 g/day. The dose of each test drug could be increased up to a maximum of 0.4 mg/day for pemafibrate or 4 g/day for DHA+EPA. In this study, medications such as statin, ezetimibe, fibrates other than pemafibrate, polyunsaturated for fatty acids other than DHA+EPA, nicotine derivatives and diabetic drugs that affect TG values, either doses were maintained unchanged or not added. In addition, all study participants received nutritional instructions from a dietician and exercise therapy instruction from a rehabilitation therapist prior to the study.

Exclusion criteria were serious liver dysfunction, gallstones, treatment with cyclosporine or rifampicin, pregnancy, and other reasons that made patients unsuitable for this study as judged by the attending physician.

This is an assessor-blinded study. The details of the study were explained to the patients, and informed consent was obtained. The study was approved by the hospital’s institutional review board (protocol no. 20181001 and approval on 22 October 2018), and the study was registered with the Hospital Medical Information Network (study ID: UMIN000034592). Study monitoring was conducted at monthly ethics committee meetings at the clinical site. The members of the ethics committee were not involved in this study.

### Endpoints

The primary endpoint was the TG level at the end of the 6-month drug administration period. The secondary endpoints were as follows: total cholesterol (T-cho), LDL, HDL, remnant-like particles cholesterol (RLP-cho), lipoprotein (a) (Lp(a)), small dense LDL (sd-LDL), fatty acid 4-fractionation (dihydro gamma-linolenic acid [DHLA], arachidonic acid [AA], EPA, DHA), EPA:AA ratio, DHA:AA ratio, blood urea nitrogen (BUN), serum creatinine (s-Cr), estimated glomerular filtration rate (eGFR), cystatin-C, urine-albumin (U-alb), HbA1c, high sensitivity C-reactive protein (hs-CRP), creatine phosphokinase (CPK), aspartate aminotransferase (AST), alanine aminotransferase (ALT), platelet count (Plt), and Fibrosis-4 (Fib-4) index. For each variable, changes at 6 months A versus pre-dose were evaluated. sd-LDL levels were estimated by Sampson’s equation using TG and LDL ^(8)^. Adverse effects were classified as renal dysfunction (increase of serum Cr ^3^ 50%), hepatic dysfunction (increase of the AST:ALT ratio ^3^ 50%), gastrointestinal symptoms, and allergic reactions. Management of adverse effects (e.g., discontinuation of the test drug) was decided by the attending physician.

### Statistical analysis

Crossover studies generally include a washout period when patients are switched to another drug. However, for ethical reasons this study did not have a washout period, so we performed a mixed effect model analysis to estimate least-squares means. The model included period, treatment, and sequence as the fixed-effect terms and patient ID as a random effect. The primary endpoint was corrected by Bonferroni. For both the first stage and the second stage, paired t-test was conducted for data at baseline.

Data were expressed as mean ± standard deviation. A p value of <0.05 was considered statistically significant.

All analyses were performed with SPSS software (SPSS Inc., Illinois, version number: 28.0.0.0, copyright holder: IBM SPSS Statistics). Data compilation was performed by Sekino Laboratory staff who were not involved in this study, and statistical analysis was supported by Data Seed Inc., a company that was not involved in conducting the study.

## Results

### Patients

Thirty-eight patients were enrolled. Baseline sociodemographic characteristics are shown in Table 1, and baseline laboratory results in Table 2.

**Table 1.** Patient Characteristics.

	Pre
Age, y	69.7 ± 18.6
Sex, male:female, n	26:10
Body mass index, kg/m^2^	24.4 ± 4.1
Diabetes mellitus	19 (53%)
Hypertension	27 (75%)
Obesity	19 (53%)
Smoking	7 (19%)
Hyperuricemia	20 (56%)
CKD (eGFR ≤59)	24 (67%)
CKD (eGFR ≤45)	11 (31%)
Ischemic heart disease	16 (44%)
Valve disease	9 (25%)
Aortic disease	7 (19%)
Cerebral infarct	3 (8%)
Peripheral atrial disease	4 (11%)

Sociodemographic characteristics of patients at baseline (N = 36). CKD: chronic kidney disease; eGFR: estimated glomerular filtration rate.

**Table 2.** Laboratory Results at Baseline (N = 36).

	First stage	Second stage	Paired t-test
Body mass index, kg/m^2^	24.4 ± 34.4	24.6 ± 34.6	0.796
Total cholesterol, mg/dL	157.3 ± 41.6	148.7 ± 28.0	0.308
LDL, mg/dL	79.7 ± 30.9	82.0 ± 25.1	0.729
HDL, mg/dL	53.6 ± 13.4	57.6 ± 16.5	0.263
sd-LDL, mg/dL	35.1 ± 12.9	29.6 13.9 ±	0.088
RLP-cho, mg/dL	6.17 ± 4.12	3.45 ± 2.42	0.002
Lp (a), mg/dL	17.2 ± 21.3	17.4 ± 16.7	0.966
DGLA, μg/dL	42.1 ± 11.1	33.7 ± 13.6	0.008
AA, μg/dL	200.2 ± 44.1	172.4 ± 50.5	0.020
EPA, μg/dL	62.2 ± 40.3	83.2 ± 65.2	0.120
DHA, μg/dL	125.9 ± 40.1	115.4 ± 34.0	0.257
EPA:AA ratio	0.32 ± 0.20	0.50 ± 0.41	0.026
DHA:AA ratio	0.65 ± 0.20	0.70 ± 0.30	0.398
High sensitivity CRP, mg/dL	0.11 ± 0.10	0.10 ± 0.92	0.725
CPK, U/L	149.3 ± 186.1	186.1 ± 268.6	0.458
BUN, mg/dL	20.7 ± 7.8	22.2 ± 8.1	0.445
Serum creatinine, mg/dL	1.05 ± 0.28	1.10 ± 0.34	0.459
eGFR, mL/min/1.73m^2^	53.3 ± 14.0	51.6 ± 16.0	0.630
Cystatin C, mg/dL	1.23 ± 1.69	1.69 ± 2.05	0.220
Urine albumin, mg/g•Cre	101.9 ± 77.0	77.0 ± 184.5	0.738
HbA1c (%)	5.66 ± 0.72	5.67 ± 0.73	0.961
AST, U/L	25.7 ± 7.7	25.1 ± 8.0	0.743
ALT, U/L	22.8 ± 18.2	18.2 ± 9.0	0.100
Platelet count, ×104μL	19.7 ± 5.4	23.8 ± 16.2	0.147
Fib-4 index	2.25 ± 1.10	2.28 ± 1.14	0.897

AA: arachidonic acid; ALT: alanine aminotransferase; AST: aspartate aminotransferase; BUN: blood urea nitrogen; CPK: creatine phosphokinase; CRP: C-reactive protein; DHA: docosahexaenoic acid; DHLA: dihydro gamma-linolenic acid; eGFR: estimated glomerular filtration rate; EPA: eicosapentaenoic acid; Fib-4: fibrosis-4; HbA1c: hemoglobin A1c; HDL: high density lipoprotein; LDL: low density lipoprotein; Lp: lipoprotein; RLP-cho: remnant like particles-cholesterol; sd-LDL: small dense low density lipoprotein.

**Figure 1.**
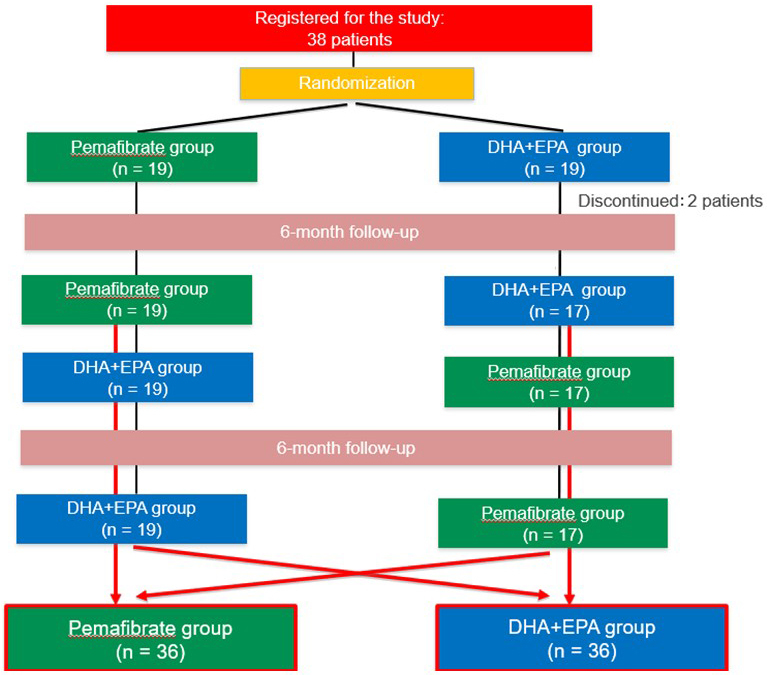
Study population.

Neither drug had any adverse effects such as renal dysfunction, hepatic dysfunction, or rhabdomyolysis. During the study, 2 adverse events were observed during treatment with DHA+EPA. One patient had difficulty swallowing and could not take a drug due to the drug presentation; the patient was free from symptoms. Another patient experienced fundus hemorrhage, and the casual relationship with DHA+EPA was unknown. Both events in these patients were Grade 1, but the study medication was discontinued. Therefore, 36 patients were included in the analysis (Figure 1). Because of the exploratory nature of this study, a formal sample size calculation was not conducted. Instead, the sample size was determined based on operational feasibility. However, to verify that the sample size of 36 patients was appropriate, the sample size calculation was conducted post-hoc, and the sample size was calculated as 16. Thus, the sample size of this study was appropriate albeit post-hoc.

All patients received oral strong statins, and 20 (56%) received ezetimibe. Seven patients (19%) received PCSK9 inhibitors. Only 3 patients were receiving statins as primary prevention, and 33 patients (92%) were receiving statins as secondary prevention. All cases achieved the LDL-C target value. Doses of pemafibrate and EPA+DHA were increased in 4 and 12 patients, respectively (p = 0.045). There were no cardiovascular or cerebrovascular events in either group during the study.

### Primary endpoint

**Figure 2.**
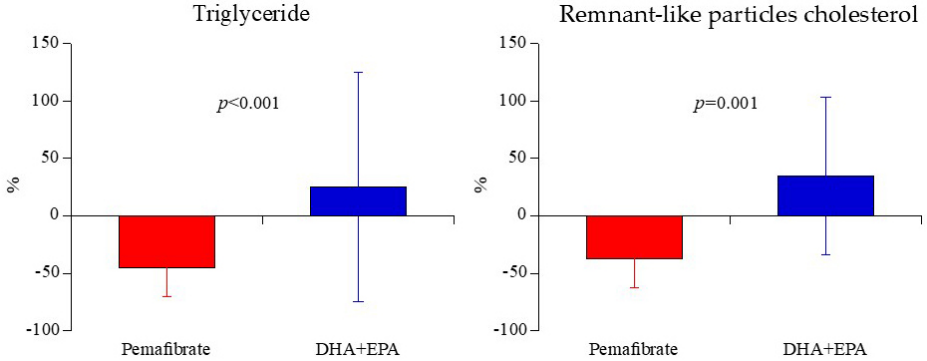
Triglyceride and remnant-like particles cholesterol levels after 6 months of treatment with pemafibrate or DHA+EPA. DHA: docosahexaenoic acid; EPA: eicosapentaenoic acid.

After 6 months of treatment, the percentage change in TG level was significantly lower in the pemafibrate group (-45.1 ± 25.6%) than in the DHA+EPA group (24.9 ± 99.7%; p < 0.001) (Figure 2). After 6 months of treatment, the absolute value in TG level was significantly lower in the pemafibrate group (95.4 ± 41.9mg/dL) than in the DHA+EPA group (146.3 ± 77.1mg/dL; p < 0.001), as well as (Table 3). The rate of achieving the target value of less than 150 mg/dL TG was also significantly higher in the pemafibrate group (94.4%) than in the DHA+EPA group (58.3%; p < 0.001).

**Table 3.** Absolute Value of Laboratory Results after 6 Months of Treatment (N = 36).

	Pemafibrate	DHA+EPA	p Value
Triglyceride, mg/dL	95.4 ± 41.9	146.3 ± 71.1	<0.001
RLP-cho, mg/dL	2.81 ± 1.91	4.46 ± 3.11	0.008
Body mass index, kg/m^2^	24.5 ± 3.6	24.3 ± 3.1	0.702 (2.81)
Total cholesterol, mg/dL	147.4 ± 28.6	149.8 ± 27.3	0.542
LDL, mg/dL	82.9 ± 24.0	79.4 ± 22.9	0.455
HDL, mg/dL	59.8 ± 16.1	54.6 ± 13.6	<0.001
sd-LDL, mg/dL	26.0 ± 8.4	29.0 ± 7.9	0.125
Lp (a), mg/dL	18.4 ± 16.9	19.4 ± 20.9	0.873
DHLA, μg/dL	39.1 ± 11.0	23.0 ± 8.8	<0.001
AA, μg/dL	175.8 ± 51.8	152.9 ± 35.6	0.003
EPA, μg/dL	52.9 ± 43.8	142.5 ± 64.5	<0.001
DHA, μg/dL	98.0 ± 31.0	143.7 ± 40.7	<0.001
EPA:AA ratio	0.29 ± 0.18	0.69 ± 0.49	<0.001
DHA:AA ratio	0.57 ± 0.22	0.96 ± 0.32	<0.001
HbA1c (%)	5.68 ± 0.90	5.61 ± 0.77	0.553
High sensitivity CRP, mg/dL	0.10 ± 0.13	0.10 ± 0.97	0.785
CPK, U/L	183.4 ± 264.6	145.1 ± 98.0	0.455
BUN, mg/dL	21.3 ± 7.7	20.7 ± 6.2	0.869
Serum creatinine, mg/dL	1.10 ± 0.34	1.08 ± 0.30	0.585
eGFR, mL/min/1.73m^2^	51.6 ± 15.1	51.9 ± 15.5	0.856
Cystatin C, mg/dL	1.39 ± 0.84	1.29 ± 0.47	0.349
Urine albumin, mg/g•Cre	72.3 ± 165.2	90.3 ± 193.4	0.634
AST, U/L	25.8 ± 9.6	26.7 ± 9.0	0.743
ALT, U/L	16.5 ± 8.8	22.0 ± 17.4	0.003
Platelet count, ×104μL	26.2 ± 15.6	21.2 ± 17.3	<0.001
Fib-4 index	2.01 ± 0.86	2.39 ± 1.17	0.001

AA: arachidonic acid; ALT: alanine aminotransferase; AST: aspartate aminotransferase; BUN: blood urea nitrogen; CPK: creatine phosphokinase; CRP: C-reactive protein; DHA: docosahexaenoic acid; DHLA: dihydro gamma-linolenic acid; eGFR: estimated glomerular filtration rate; EPA: eicosapentaenoic acid; HbA1c: hemoglobin A1c; HDL: high density lipoprotein; LDL: low density lipoprotein; Lp: lipoprotein; RLP-cho: remnant like particles-cholesterol; sd-LDL: small dense low density lipoprotein.

### Secondary endpoints

Secondary endpoint results in each group are shown in Table 3 and 4.

**Table 4.** Change Rate of Laboratory Results after 6 Months of Treatment (N = 36).

	Pemafibrate	DHA+EPA	p Value
Body mass index, kg/m^2^	1.2 ± 4.2	0.7 ± 2.8	0.694
Total cholesterol, mg/dL	-2.3 ± 14.4	2.5 ± 16.4	0.284
LDL, mg/dL	7.2 ± 26.1	3.5 ± 29.4	0.649
HDL, mg/dL	10.3 ± 13.2	-1.7 ± 14.4	<0.001
sd-LDL, mg/dL	-19.3 ± 20.5	4.2 ± 36.6	0.001
Lp (a), mg/dL	17.3 ± 38.2	36.9 ± 62.7	0.898
DHLA, μg/dL	30.9 ± 73.5	-37.9 ± 26.5	<0.001
AA, μg/dL	-3.4 ± 24.7	-15.5 ± 13.0	0.003
EPA, μg/dL	-18.3 ± 72.8	196.1 ± 137.7	<0.001
DHA, μg/dL	-23.8 ± 22.4	37.5 ± 38.5	<0.001
HbA1c (%)	-0.5 ± 6.0	-.0.02 ± 9.4	0.720
High sensitivity CRP, mg/dL	12.5 ± 85.3	25.7 ± 98.5	0.794
CPK, U/L	16.0 ± 72.0	8.5 ± 40.1	0.705
BUN, mg/dL	3.9 ± 25.0	1.0 ± 24.6	0.934
Serum creatinine, mg/dL	4.2 ± 13.6	1.9 ± 10.1	0.282
eGFR, mL/min/1.73m^2^	-2.7 ± 15.6	-1.3 ± 10.5	0.324
Cystatin C, mg/dL	8.3 ± 23.3	-5.1 ± 19.8	0.127
Urine albumin, mg/g•Cre	74.7 ± 168.2	194.2 ± 826.1	0.452
AST, U/L	0.9 ± 25.0	10.4 ± 30.3	0.221
ALT, U/L	-21.5 ± 25.7	31.3 ± 62.6	<0.001
Platelet count, ×104μL	18.2 ± 54.6	-7.1 ± 12.4	0.022
Fib-4 index	-2.3 ± 14.7	11.9 ± 22.4	0.031

AA: arachidonic acid; ALT: alanine aminotransferase; AST: aspartate aminotransferase; BUN: blood urea nitrogen; CPK: creatine phosphokinase; CRP: C-reactive protein; DHA: docosahexaenoic acid; DHLA: dihydro gamma-linolenic acid; eGFR: estimated glomerular filtration rate; EPA: eicosapentaenoic acid; HbA1c: hemoglobin A1c; HDL: high density lipoprotein; LDL: low density lipoprotein; Lp: lipoprotein; RLP-cho: remnant like particles-cholesterol; sd-LDL: small dense low density lipoprotein.

**Figure 3.**
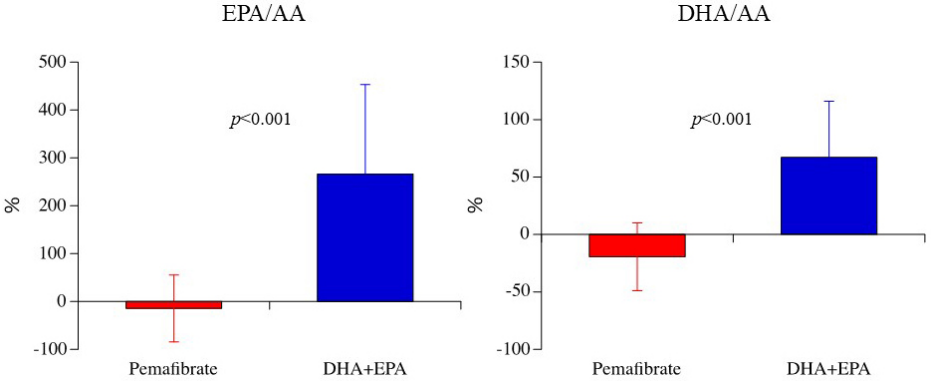
Ratio of eicosapentaenoic acid to arachidonic acid and of docosahexaenoic acid to arachidonic acid after 6 months of treatment with pemafibrate or DHA+EPA. DHA: docosahexaenoic acid; EPA: eicosapentaenoic acid.

In the change rate of lipid-related biomarkers after 6 months of treatment, there were no significant difference between the 2 groups in T-cho, LDL, or Lp (a), but HDL was significantly higher in the pemafibrate group than in the DHA+EPA group. After 6 months of treatment, the percentage change in RLP-cho level was significantly lower in the pemafibrate group (-37.5 ± 24.7%) than in the DHA+EPA group (34.9 ± 68.5%; p = 0.001) (Figure 2). Similarly, in absolute values, the RLP-cho level was significantly lower in the pemafibrate group (2.81 ± 1.91 mg/dL) than in the DHA+EPA group (4.46 ± 3.11 mg/dL; p = 0.008). After 6 months of treatment, the percentage change in sd-LDL levels was significantly lower in the pemafibrate group (-19.3 ± 20.5%) than in the DHA+EPA group (4.2 ± 36.6%; p = 0.001). In terms of absolute values, there was no significant difference in sd-LDL levels between the 2 groups (p = 0.125). Regarding the change rate of fatty acid-related biomarkers after 6 months of treatment, the DHLA and AA levels were significantly higher in the pemafibrate group than in the DHA+EPA group, while the EPA and DHA levels were significantly lower in the pemafibrate group than in the DHA+EPA group. After 6 months of treatment, the percentage change in EPA:AA ratio and DHA:AA ratios were significantly higher in the DHA+EPA group (266.6 ± 186.6% and 67.2 ± 48.9%, respectively) than in the pemafibrate group (-14.8 ± 69.8% and -19.4 ± 29.5%, respectively; both p < 0.001) (Figure 3). Similarly, in terms of the absolute values of fatty acids (DHLA, AA, EPA, and DHA), after 6 months of treatment, the absolute value in EPA:AA ratio and DHA:AA ratios were significantly higher in the DHA+EPA group (0.69 ± 0.49 and 0.96 ± 0.32, respectively) than in the pemafibrate group (0.29 ± 0.18 and 0.57 ± 0.22, respectively; both p < 0.001).

Regarding the change rate and absolute value of kidney-related biomarkers after 6 months of treatment, there were no significant difference between the 2 groups for BUN, s-Cr, eGFR, cystatin-C, and U-alb.

In the change rate and absolute value of liver-related biomarkers after 6 months of treatment, there were no significant difference in AST between the 2 groups, but ALT was significantly lower in the pemafibrate group than in the DHA+EPA group (change rate: p < 0.001, absolute value: p = 0.003).

In the change rate and absolute value of other biomarkers after 6 months of treatment, there were no significant differences in HbA1c, hs-CRP, or CPK between the 2 groups, but the platelet count was significantly higher in the pemafibrate group than in the DHA+EPA group (change rate: p = 0.022, absolute value: p < 0.001), and the Fib-4 index was significantly lower in the pemafibrate group than in the DHA+EPA group (change rate: p = 0.031, absolute value: p = 0.001).

## Discussion

This study showed that TG levels decreased significantly more with pemafibrate than with DHA+EPA. Furthermore, compared with DHA+EPA, pemafibrate significantly increased HDL and decreased RLP-cho. In contrast, in all categories of fatty acid 4-fractionation, DHA+EPA showed significantly better results than pemafibrate. Furthermore, Fib-4 index, an index for liver fibrosis, was significantly better with pemafibrate than DHA+EPA. Based on these results, it was shown that pemafibrate is an effective first-line therapy for hypertriglyceridemia. DHA+EPA also demonstrated that it is beneficial for fatty acids compared to pemafibrate. Thus, when pemafibrate is not effective enough in improving the fatty acids profile, the addition of DHA+EPA is considered useful.

Various international guidelines for preventing arteriosclerosis clearly state that high TG levels increase the risk of developing cardiovascular diseases such as coronary artery disease. LDL-lowering therapy with statins has been proven to prevent cardiovascular disease ^(9), (10), (11)^. However, even if LDL is strictly controlled at 70 mg/dL, the risk for cardiovascular events increases if the TG level is 200 mg/dL or higher ^(11)^, making it important to also lower TG and not only LDL. While there have been numerous reports on the effect of statins in reducing cardiovascular events, there have also been reports on the residual risk after LDL-lowering therapy ^(12), (13), (14), (15)^, and high TG and low HDL have been reported to be associated with residual risk ^(16), (17)^. Although our study was a short-term crossover study and did not examine cardiovascular events, all patients in our study were treated with statins, and the fact that pemafibrate significantly improved TG and HDL compared to DHA+EPA is meaningful.

High RLP-cho is also an independent risk factor even when LDL is maintained at a level below 100 mg/Dl ^(18)^. RLP-cho, like LDL, is an atherosclerosis-inducing factor and is strongly involved in the development of cardiovascular events, and it has been reported that statins, as well as fibrates, effectively reduce RLP-cho ^(19), (20)^. In our study, RLP-cho was proven to be significantly reduced by pemafibrate compared to DHA+EPA, and we believe that pemafibrate is an important drug to lower RLP-cho together with TG. Although the TG-lowering and HDL-increasing effects of fibrate drugs have been recognized, when used in combination with statins they can cause renal dysfunction and rhabdomyolysis. The selective PPARα modulator pemafibrate evaluated in this study has minimal effects on the liver and kidneys and is, therefore, approved for use in combination with statins. The results of this study showed that pemafibrate has a strong TG-lowering effect and has no effect on the liver or kidneys, making it a promising drug for concomitant use with statins. In a study of 32 Japanese patients with hypertriglyceridemia, it was reported that pemafibrate significantly reduced TG and RLP-cho without worsening renal or hepatic function ^(21)^. In a study of 39 Japanese patients with hypertriglyceridemia and chronic kidney disease, it was reported that pemafibrate did not worsen renal function, and that a change of treatment from fenofibrate or bezafibrate to pemafibrate resulted in an improvement in renal function ^(22)^. A large study of pemafibrate in 10,497 patients with type 2 diabetes mellitus-associated hypertension with hypertension (PROMINENT study) showed that pemafibrate had a strong TG and RLP-cho lowering effect and HDL-raising effect; however, LDL was increased, and the primary endpoint (nonfatal myocardial infarction, ischemic stroke, coronary revascularization, and cardiovascular death) was reported to be no different from the placebo group [6]. The results of this study do not rule out the possibility that the increase in LDL-C levels observed in the pemafibrate group counteracted the benefit of lower TG and remnant cholesterol levels, indicating the complexity of lipid indicators as a residual risk. In addition, 96% of the patients in this study received statins, and LDL was controlled to 70 mg/dL before the study began. Furthermore, the fact that angiotensin converting enzyme inhibitors/ angiotensin receptor blockers, which have been shown to be effective in many large-scale trials, were administered to 80% of patients, SGLT2 inhibitors to 17% of patients, and GLP-1 receptor agonists to 10% of patients is likely to have had an impact. The fact that pemafibrate was administered to 80% of the patients in the PROMINENT study is also considered a reason why pemafibrate could not be shown to be effective in that study. However, an interesting sub-analysis of the PROMINENT trial reported that the incidence of lower limb ischemic ulcers or gangrene was statistically significantly lower in the pemafibrate group than in the placebo group ^(23), (24)^. The result of this study demonstrated potent TG-lowering effects, RLP-cho lowering effects, as well as improvement in liver fibrosis. Therefore, it is considered a worthwhile further investigation. sd-LDL refers to small dense LDL particles, which are more likely to accumulate in the vascular wall and have been reported to be associated with coronary artery disease ^(25)^. In the PROMINENT trial, although TG levels decreased with the addition of pemafibrate, sd-LDL did not decrease ^(6), (26)^. However, the PRESTAGE study, which included a total of 97 patients with type 2 diabetes and hypertriglyceridemia who were treated with statins, randomly assigned participants to receive either add-on pemafibrate (0.2 mg/day) or doubling of the statin dose. The study showed that the percentage and absolute reductions in sd-LDL levels were significantly greater in the pemafibrate add-on group than in the statin-doubling group (-32.8% vs. -8.1%; -16 vs. -3 mg/dL, respectively). TG levels were reduced only in the pemafibrate add-on group (-44%), whereas LDL levels were reduced only in the statin-doubling group (-8%) ^(27)^. In our study, although there was no difference in absolute values (p = 0.125), the percentage change showed a significant reduction with pemafibrate compared to DHA+EPA. Since the subjects of our study had hypertriglyceridemia and lower LDL, but not markedly low HDL, compared with those in the PROMINENT trial, and because 19% of patients received PCSK9 inhibitors and all cases achieved the LDL target value, pemafibrate demonstrated potential efficacy in reducing sd-LDL, particularly when LDL and HDL were already within therapeutic ranges. As our study was short-term, long-term observation may reveal further beneficial effects on cardiovascular events.

In the Reduction of Cardiovascular Events with Icosapent Ethyl-Intervention Trial (REDUCE-IT) trial, which observed the efficacy of EPA in 8,179 patients with hypertriglyceridemia and low HDL levels who were taking statins, it was reported that the primary endpoint (a composite of cardiovascular death, nonfatal myocardial infarction, nonfatal stroke, coronary revascularization, and unstable angina) was significantly reduced compared to placebo ^(28)^. In the Japan EPA Lipid Intervention (JELIS) trial, which examined whether EPA was effective in preventing coronary artery events in 18,645 Japanese hyperlipidemic patients taking statins with an average observation period of 4.6 years, the efficacy of the drug in preventing coronary artery disease was demonstrated. In the JELIS trial, it was also shown that, in addition to lowering TG, the AA ratio (EPA:AA ratio) was significantly increased compared to baseline (0.63 to 1.23 after EPA administration) ^(29), (30)^. The results of this clinical study demonstrated the importance of managing TG and fatty acids as well as LDL. However, some clinical studies on EPA have not shown any efficacy, and Nishizaki et al. ^(31)^ suggested that the reason for this is because the studies that showed good results used high-dose EPA, had a high EPA:AA ratio at baseline, and the studies that showed poor results used low-dose EPA and had a low EPA:AA ratio at baseline. The fatty acid values and EPA:AA ratios that should be treated are not currently clear. Furthermore, there are few comparative studies between fibrates, drugs that lower TG, and n-3 fatty acids, and the characteristics and efficacy of each drug have not yet been fully evaluated and examined. To date, few studies have compared pemafibrate with EPA+DHA preparations. However, the results of this study showed that, after treatment, the EPA:AA ratio was 0.69 ± 0.49 and the DHA:AA ratio was 0.96 ± 0.32 in the EPA+DHA group, whereas in the pemafibrate group, the EPA:AA and DHA:AA ratios were 0.29 ± 0.18 and 0.57 ± 0.22, respectively. These values were significantly higher in the EPA+DHA group than in the pemafibrate group, indicating that EPA+DHA had better outcomes than pemafibrate in terms of fatty acid 4-fractionation. Similarly to the present study there is a report of a prospective, multicenter, open-label, randomized, parallel group trial in Japanese patients with dyslipidemia receiving statin treatment comparing the effects of pemafibrate and DHA+EPA) (PROUD28 study) ^(32)^. The PROUD28 study reported that pemafibrate significantly decreased apoB-48, TG, RLP-cho, apoC-III, ALP, gamma-glutamyl transpeptidase, and alkaline phosphatase compared to DHA+EPA, while HDL, apoA-I, and apoA-II significantly increased. Based on the results of the PROUD28 study and our research, pemafibrate significantly reduced TG and RLP-cho compared to DHA+EPA, and increased HDL. These findings clearly indicate that pemafibrate reduces the residual risk of atherosclerotic vascular diseases compared to DHA+EPA.

Some clinical studies have shown that EPA preparations prevent cardiovascular events ^(24), (25), (26)^, and a few studies found that DHA+EPA or DHA preparations are effective ^(1), (33)^. In patients undergoing percutaneous coronary intervention, a high EPA:AA ratio was reported to be associated with a reduction in events after the intervention and the DHA:AA ratio was not associated with the occurrence of events ^(34)^. Seko et al. ^(35)^ performed a detailed analysis of fatty acids in their research on the effects of pemafibrate on fatty acids and reported that pemafibrate both lowers TG and improves total fatty acids, saturated fatty acids, monounsaturated fatty acids, polyunsaturated fatty acids (PUFA), n-3 PUFA, and n-3 PUFA.

In our study, although pemafibrate had a weaker positive effect on fatty acids than EPA+DHA did, it did not worsen omega-6 unsaturated fatty acids (DHLA, AA); however, further investigation is needed in the future. Recent studies have investigated the efficacy of pemafibrate in metabolic dysfunction-associated steatotic liver disease (MASLD) ^(36)^. In a clinical study of patients with type 2 diabetes mellitus complicated with MASLD whose ALT did not normalize, pemafibrate administration was reported to normalize markers of hepatic inflammation, function, and fibrosis, including ALT and Fib-4 index ^(37)^. In a double-blind, placebo-controlled, randomized multicenter, phase 2 trial of pemafibrate in patients with MASLD, pemafibrate was associated with improvement in ALT. In a double-blind, placebo-controlled, randomized multicenter, phase 2 trial of pemafibrate in patients with MASLD, significant improvement in liver stiffness by magnetic resonance elastography was reported compared with placebo, as well as improvement in ALT ^(38)^. Although we believe that MASLD patients are included in our study, they have not been diagnosed with MASLD, pemafibrate significantly improved ALT and Fib-4 index compared to DHA+EPA, and this is an area for further investigation. In a subgroup analysis of the PROUD28 study, pemafibrate significantly reduced the fatty liver index (FLI) compared with DHA+EPA, suggesting that pemafibrate may be an effective drug for the treatment of hypertriglyceridemia and reduction of the risk of MASLD ^(39)^. FLI is an index derived from BMI, waist circumference, γ-GTP, and TG, and is considered useful for the diagnosis of MASLD/Non-alcoholic fatty liver disease (NAFLD) (C). In our study, we were unable to show FLI because waist circumference and γ-GTP could not be measured, but we consider the results of the subgroup analysis of the PROUD28 study to be meaningful ^(40)^. In a comparative study with omega-3-acid ethyl ester, Sumida et al. ^(41)^ reported that AST and hepatic fibrosis biomarkers (Mac-2 binding protein glycan isomer, Fib-4 index) were significantly improved with pemafibrate compared with omega-3-acid ethyl ester and that pemafibrate is preferable to omega-3 fatty acid ethyl ester for lipid management and treatment of MASLD patients with hypertriglyceridemia. In the present study, patients did not have a diagnosis of MASLD, but similar to the results of the study by Sumida et al. ^(41)^, our results showed that AST and Fib-4 index were significantly lower with pemafibrate than with omega-3-acid ethyl ester. We believe that in the future, pemafibrate can be expected to be used not only for the treatment of hypertriglyceridemia but also for the treatment of MASLD.

There are clinical studies that have proven the efficacy of pemafibrate and DHA+EPA as drugs that lower TG. However, there are few clinical studies that have compared the 2 groups, and we believe that there is value in conducting this study. TG has been proven to be a residual risk in statin treatment. From the results of our study, we found that the TG-lowering effect of pemafibrate is higher than that of DHA+EPA. However, we also found that DHA+EPA has a greater effect on fatty acids than pemafibrate. Orally administered EPA is primarily incorporated into (1) cell membrane phospholipids, (2) blood lipid fractions (phospholipids, neutral lipids, and cholesteryl esters), and (3) adipose tissue triacylglycerols, and in the liver, it is involved in metabolism and esterification in microsomes and mitochondria. EPA and DHA are stored in adipose tissue, liver, and muscle. Some basic studies have shown that EPA is particularly long-lasting in adipose tissue, with half-lives ranging from 6 months to 2 years ^(42), (43), (44)^. Because this study compared the 2 drugs in a crossover design, it is unclear whether EPA had disappeared from intracellular storage sites by 6 months after crossover from DHA+EPA to pemafibrate. However, we believe, based on blood test data at month 6 after switching from DHA+EPA to pemafibrate, that fatty acid disappeared.

Our results suggest that lowering TG, which has been proven to be a residual risk, should be a priority, and that in cases where TG is lowered but EPA:AA ratio is low, the combination of DHA+EPA may also be necessary. Although TG has a treatment target value set in the guidelines, this is not the case for fatty acids. We believe that clarifying the treatment target values for fatty acids will help to further reduce cardiovascular events, and we look forward to future clinical research on fatty acids.

### Conclusions

This study showed that when used in combination with a statin, pemafibrate had a stronger effect on lowering TG and RLP-cho than DHA+EPA and did not cause adverse effects such as renal dysfunction or rhabdomyolysis. In contrast, DHA+EPA was found to be more effective than pemafibrate in terms of fatty acids. The results indicate that pemafibrate may be a good first choice for hypertriglyceridemia and that add-on DHA+EPA can be considered if pemafibrate is not sufficiently effective. However, these results need to be confirmed in larger samples in the future. Moreover, to better understand the characteristics of each drug, we did not evaluate patients treated with both pemafibrate and DHA+EPA. The study result indicates a need to investigate a combination of pemafibrate and DHA+EPA to exploit the advantages of each drug. Along these lines, we will study patients on both pemafibrate and DHA+EPA in the future.

### Limitations

There are several limitations of this study. First, this was an exploratory study and was conducted at a single site with a limited number of study subjects. Second, a washout period is usually established when a drug is switched to another in a crossover study design. However, due to ethical reasons, a washout period was not established in this study. To compensate for the carry-over effects of a prior drug, efficacy was evaluated at the end of the 6-month treatment period. Third, the pemafibrate used in this study was a tablet formulation, while DHA+EPA was a granular capsule; hence, a single formulation cannot be used. A pharmacist monitored medication adherence and confirmed that all patients took medication. However, it cannot be denied that a difference in the formulations might have affected the study results.

## Article Information

### Acknowledgments

We would like to express our sincere appreciation to Kazuaki Obata, Yoshitarou Shimizu, and Sakie Kanno, Medical Technologists at Sekino Hospital, for their constructive cooperation.

### Author Contributions

Akira Sezai: Conceptualization, methodology, validation, formal analysis, investigation, resources, data curation, writing, original draft preparation, supervision, project administration.

Makoto Taoka: Conceptualization, validation, investigation, resources, review, and editing.

Hisakuni Sekino: Conceptualization, validation, review and editing, funding acquisition.

Masashi Tanaka: Conceptualization, methodology, validation, review, and editing.

All authors have read and agreed to the published version of the manuscript. Authorship must be limited to those who have contributed substantially to the work reported.

### Conflicts of Interest

None

### IRB Approval Code and Name of the Institution

Protocol no. 20181001, Sekino Hospital.
